# Associations between Organochlorine Pesticides and Vitamin D Deficiency in the U.S. Population

**DOI:** 10.1371/journal.pone.0030093

**Published:** 2012-01-25

**Authors:** Jin-Hoon Yang, Yu-Mi Lee, Sang-Geun Bae, David R. Jacobs, Duk-Hee Lee

**Affiliations:** 1 Department of Preventative Medicine, School of Medicine, Kyungpook National University, Daegu, Korea; 2 Department of Occupational Medicine, CHA Gumi Medical Center, CHA University, Gumi, Korea; 3 Division of Epidemiology, School of Public Health, University of Minnesota, Minneapolis, Minnesota, United States of America; 4 Department of Nutrition, University of Oslo, Oslo, Norway; Brigham & Women's Hospital, and Harvard Medical School, United States of America

## Abstract

**Background:**

Recently low dose organochlorine (OC) pesticides have been strongly linked to various chronic diseases including diabetes and cardiovascular diseases. Both field and animal studies have suggested a possibility that persistent lipophilic chemicals like OC pesticides can cause vitamin D deficiency, but there have been no human studies of exposure to any chemical as a possible cause of vitamin D deficiency. This study was performed to examine if serum concentrations of OC pesticides were associated with serum concentrations of 25-hydroxyvitamin D (25(OH)D) in the U.S. general population.

**Methodology/Principal Findings:**

Cross-sectional associations of serum OC pesticides with serum 25(OH)D were investigated in 1,275 subjects aged ≥20 in the National Health and Nutrition Examination Survey(NHANES), 2003–2004. We selected 7 OC pesticides detectable in ≥80% of participants. Among the 7 OC pesticides, *p,p′*-DDT (β = −0.022, P<0.01), *p,p′*-DDE (β = −0.018, P = 0.04), and β-hexachlorocyclohexane (β = −0.022, P = 0.02) showed significant inverse associations with serum 25(OH)D levels. When study subjects were stratified by age, race, and the presence of various chronic diseases, *p,p′*-DDT showed consistent inverse associations in all subgroups, although stronger associations tended to be observed among subjects with old age, white race, or chronic diseases.

**Conclusion/Significance:**

The current study suggests that the background exposure to some OC pesticides leads to vitamin D deficiency in human. Considering the importance of vitamin D deficiency in the development of chronic diseases, chemical exposure as a possible cause of vitamin D deficiency should be evaluated in prospective and experimental studies.

## Introduction

In the past, the major health problems resulting from vitamin D deficiency were rickets in children and osteomalacia in adults [1]. Recently there has been intense interest in the role of vitamin D in a variety of nonskeletal medical conditions. Indeed, vitamin D deficiency has been associated with increases in cardiovascular disease, cancer, and infection [1]. At present, despite controversy over the definition of vitamin D deficiency, insufficient serum vitamin D levels is very common in the general population [2], [3]. Recently, vitamin D supplementation has been recommended and widely applied with the intention of preventing many diseases related to vitamin D deficiency [4], [5].

Sunlight exposure is the primary determinant of vitamin D status in humans. Therefore, the control against sun exposure through avoidance and sun protection has been regarded as a main cause of increased prevalence of vitamin D deficiency [6]. In addition, decreased outdoor activity and obesity has been also associated with vitamin D deficiency [6].

To our best knowledge, there have been no studies of exposure to any chemical as a possible cause of vitamin D deficiency in human. However, there was an interesting field study which observed clear inverse associations of hepatic load of DDT and polychlorinated biphenyls (PCBs) with circulating 1,25-dihydroxyvitamin D_3_ (1,25(OH)_2_D) status in grey seals in the Baltic Sea [7]. DDT and PCBs are chemicals which are Persistent Organic Pollutants (POPs). POPs include a variety of chemicals such as polychlorinated dibenzo-*p*-dioxins (PCDDs), polychlorinated dibenzofurans (PCDFs), PCBs, and organochlorines (OC) pesticides, and have common properties like resistance to environmental degradation, lipophilicity, and bioaccumulation in food chain. Supporting the observation from the field study, a couple of animal studies reported that the exposure to PCBs decreased levels of 25-hydroxyvitamin D (25-(OH)D) and 1,25(OH)_2_D [8], [9].

In fact, there has been emerging evidence strong relations between the background exposure to POPs and various diseases including type 2 diabetes and cardiovascular diseases [10], [11], [12], [13]. As well-known endocrine disruptors, it is possible that exposure to POPs can affect metabolism of vitamin D, because vitamin D is a hormone.

This study was performed to examine if serum concentrations of POPs were associated with serum concentrations of 25-hydroxyvitamin D (25-(OH)D) in the U.S. general population, as represented in subsamples of the National Health and Examination Survey (NHANES) 2003–2004. Although we examined various kinds of POPs, there was no reportable association with POPs belonging to subclasses of PCDDs, PCDFs, and PCBs. As only POPs belonging to OC pesticides showed interesting associations with serum concentrations of 25-(OH)D, we report results on OC pesticides here.

## Materials and Methods

### Study subjects

The National Health and Nutrition Examination Survey (NHANES) conducted by the Centers for Disease Control and Prevention (CDC) was designed to be nationally representative of the non-institutionalized U.S. population. This report is a cross-sectional study using the NHANES data 2003–2004 collected between January 2003 and December 2004. For NHANES 2003–2004, 12,761 persons were selected for the sample, 10,122 of those were interviewed, and 9,643 were examined in the Mobile Examination Center (MEC). OC pesticides were measured in 2,337 subsamples of persons 12 years and over. Among 1,585 persons aged 20 or more, the final sample size was 1,275 after excluding pregnant women and subjects without information on OC pesticides and serum vitamin D levels. The study protocol was reviewed and approved by the CDC institutional review board; additionally, informed written consent was obtained from all subjects before they took part in the study.

### Measurements

Venous blood samples were collected in the MEC and shipped weekly to laboratories at −20°C. Thirteen organochlorine pesticides were measured in serum by high-resolution gas chromatography/isotope-dilution high-resolution mass spectrometry (HRGC/ID-HRMS). We selected the 7 organochlorine pesticides for which at least 80% of study subjects had concentrations>LOD; p,p′-DDE, p,p′-DDT, β-hexachlorocyclohexane, Dieldrin, Hexachlorobenzene, Oxychlordane, and Trans-nonachlor.

Serum 25-(OH)D was measured by the Diasorin 25-(OH)D assay, which consists of a two-step procedure. The first procedure is an extraction of 25-(OH)D and other hydroxylated metabolites from serum, followed by an equilibrium Radioimmunoassay (RIA) procedure based on a specific antibody to 25-(OH)D.

### Statistical analyses

Adjusted means of serum 25-(OH)D levels across 5 categories of serum concentrations of POPs were examined by General Linear Models. Serum concentrations of OC pesticides were categorized into quintiles and the last quintile was further divided into two groups using the median values of each OC pesticides as OC pesticides showed very right-skewed distributions. We also used summary measure of OC pesticides by summing individual rank of each OC pesticide and the sum was categorized using the same method as with the individual OC pesticides. Serum 25-(OH)D levels were log transformed because they also showed a right-skewed distribution. In addition, we used General Additive Models to check the possibility of a non-linear spline across the whole range of serum concentrations of OC pesticides.

Adjusting variables were sex, age (years), race, body mass index (continuous), smoking status (never, former, current), outdoor activities (continuous, total frequency of walked or bicycled as part of getting to and from work, or school, or to do errands per day during recent 1 month), and vitamin D supplementation (continuous, total amount of vitamin D intake by supplement during recent 1 month).

As POPs levels were very different depending on age or race, stratified analyses by age (<60 vs. ≥60 years) or race (white vs. non-white) were performed to see if there were consistent patterns. In addition, we also performed stratified analyses by the status of presence of self-reported common chronic diseases (coronary heart diseases, angina pectoris, congestive heart failure, stroke, emphysema, bronchitis, chronic liver diseases, thyroid diseases, cancer, hypertension, diabetes, or kidney diseases) because serum concentrations of vitamin D are associated with various clinical conditions.

All statistical analyses were performed with SAS 9.1 and SUDAAN 9.0. Estimates of main results were calculated accounting for stratification and clustering [14], adjusting for age, race and ethnicity, and poverty income ratio instead of using sample weights; this adjustment has been regarded as a good compromise between efficiency and bias [14], [15]. As results were very similar with SAS 9.1 and SUDAAN 9.0, we present the results based on SAS 9.1.

## Results

The sample of 1,275 participants included 50.1% men and 52.9% non-Hispanic white. Table 1 summarizes the distribution of demographics, health behaviors, and other characteristics by quintiles of p,p′-DDT, one of OC pesticides which showed strongest associations with serum 25-(OH)D level. Subjects with high serum concentrations of p,p′-DDT were older, more obese, non-white race, and non-smoker, compared with those with low serum concentrations of p,p′-DDT. They had also higher prevalence of hypertension or diabetes. Other OC pesticides showed similar associations with covariates as did p,p′-DDT (data not shown).

**Table 1:** Distribution of demographic, and other characteristics by quintiles of p,p′-DDT.

	Quintiles of p,p′-DDT			
	Q1	Q3	Q5	P_trend_
Mean±standard deviation				
Age (years)	41.6±17.9	49.6±18.3	62.0±16.8	<0.01
BMI (kg/m^2^)	26.9±5.8	28.5±5.7	29.1±6.3	<0.01
Vitamin D supplement (IU)*	1082±3247	991±3119	902±3250	0.36
Outdoor activities (times/ day)*	4.2±12.6	5.9±19.9	4.6±12.5	0.28
Percent (%)				
Men	45.5	53.9	43.7	0.71
White race	66.2	54.7	29.5	<0.01
Current smoker	26.1	21.5	12.2	<0.01
Vitamin D supplement user	11.7	12.6	10.2	0.70
Hypertension	13.6	23.1	38.8	<0.01
Diabetes	2.0	9.3	22.1	<0.01

*Vitamin D supplement: total amount of vitamin D intake by supplement during recent 1 month; outdoor activities: total frequency of walked or bicycled as part of getting to and from work, or school, or to do errands per day during recent 1 month.

Among 7 OC pesticides, p,p′-DDT, p,p′-DDE, and β-hexachlorocyclohexane showed significant inverse associations with serum 25-(OH)D (P<0.01) (Table 2). Whenever there were significant associations, the decreases of serum 25-(OH)D levels were particularly prominent in the last category of OC pesticides, belonging to the highest 10% of study subjects. Based on beta-cofficients, the stronger associations were observed with p,p′-DDT and β-hexachlorocyclohexane rather than p,p-DDE. When we used the summary measure of 3 OC pesticides which showed significant inverse associations, the strength of association seemed to be a little stronger than individual OC pesticides.

**Table 2:** Adjusted* geometric means (standard error) of serum 25-hydroxyvitamin D (25(OH)D) levels (ng/ml) by categories of serum organochlorine (OC) pesticides levels.

	Quintiles of each OC pesticides							
	Q1	Q2	Q3	Q4	Q5			
					<median	≥median		
Concentrations of each OC pesticides (ng/g lipid)								
*p,p′*-DDE	≤120	121–238	239–491	492–1120	1121–2010	2010		
*p,p′*-DDT	≤3.0	3.1–4.4	4.5–6.3	6.4–11.2	11.3–19.8	>19.8		
β-hexachlorocyclohexane	≤3.0	3.1–6.4	6.5–13.7	13.8–29.7	29.8–53.9	>53.9		
Diedrin	≤4.0	4.1–5.9	6.0–7.8	7.9–11.3	11.4–15.8	>15.8		
Hexachlorobenzene	≤11.5	11.6–14.4	14.5–17.4	17.5–22.0	22.1–26.2	>26.2		
Oxychlordane	≤5.0	5.1–10.4	10.5–17.1	17.2–27.6	27.7–38.1	>38.1		
Trans-nonachlor	≤8.4	8.5–15.3	15.4–27.6	27.7–46.1	46.2–69.8	>69.8		
Adjusted* geometric means of 25(OH)D (ng/ml)							β	P_trend_
*p,p′*-DDT	21.9(0.6)	20.7(0.5)	20.1(0.5)	20.2 (0.5)	20.9(0.8)	18.7 (0.7)	−0.022	<0.01
*p,p′*-DDE	20.6(0.6)	21.1(0.5)	21.8(0.5)	19.8(0.5)	19.9(0.7)	18.9(0.7)	−0.018	0.04
β-hexachlorocyclohexane	21.1(0.6)	20.4(0.5)	21.3(0.5)	20.4(0.5)	20.7(0.8)	18.1(0.7)	−0.022	0.02
Diedrin	21.2(0.6)	20.9(0.5)	20.1(0.5)	21.0(0.5)	18.8(0.7)	20.3(0.8)	−0.014	0.10
Hexachlorobenzene	20.1(0.5)	20.0(0.5)	20.8(0.5)	20.7(0.5)	21.7(0.8)	20.4(0.8)	0.010	0.19
Oxychlordane	20.9(0.6)	20.6(0.6)	20.8(0.5)	20.5(0.5)	20.7(0.8)	19.0(0.8)	−0.013	0.23
Trans-nonachlor	20.7(0.6)	20.5(0.5)	20.2(0.5)	21.2(0.6)	20.5(0.8)	19.8(0.8)	−0.004	0.69
Sum of 3 OC pesticides†	21.3(0.6)	21.0(0.5)	20.8(0.5)	20.4(0.5)	19.9(0.7)	18.4(0.7)	−0.025	<0.01
Sum of 7 OC pesticides‡	20.8(0.6)	20.8(0.6)	21.3(0.5)	20.2(0.5)	19.8(0.8)	19.2(0.8)	−0.016	0.11

*Adjusted for gender, age, race, BMI, smoking status, outdoor activity, and vitamin D supplement.

† The summary measure of 3 OC pesticides was calculated by summing individual ranks of 3 OC pesticides (p,p′-DDE, p,p′-DDT, and β-hexachlorocyclohexane).

‡ The summary measure of all 7 OC pesticides was calculated by summing individual ranks of 7 OC pesticides.

When study subjects were stratified by age, race, and the presence of various chronic diseases, p,p′-DDT showed consistent inverse associations in all subgroups (Table 3). However, stronger associations tend to be observed among subjects at least 60 years old or having chronic disease, although none of the p values for interaction reached statistical significance. Similar to the results for p,p′-DDT, both p,p-DDE and β-hexachlorocyclohexane showed significant or marginally significant inverse associations with serum 25-(OH)D levels only among older subjects or those with chronic disease.

**Table 3:** Adjusted* geometric means (standard error) of serum 25-hydroxyvitamin D (25(OH)D) levels (ng/ml) by categories of serum organochlorine (OC) pesticides levels after stratifying by age, race, or chronic disease status.

	Quintiles of each OC pesticides								
	Q1	Q2	Q3	Q4	Q5				
					<median	≥median	β	P_trend_	P_interaction_
*p,p′*-DDT									
Age<60 (n = 819)	22.0(0.6)	20.5(0.6)	19.5(0.6)	20.0(0.6)	20.8(1.1)	20.2(1.2)	−0.019	0.06	0.48
Age≥60 (n = 456)	21.7(1.3)	21.5(1.1)	21.8(1.0)	20.5(0.8)	20.8(1.0)	17.6(0.9)	−0.036	<0.01	
White race (n = 675)	25.5(0.9)	24.5(0.8)	24.6(0.6)	22.8(0.6)	24.1(0.9)	21.8(0.7)	−0.025	0.03	0.43
Other races (n = 600)	18.7(0.8)	16.9(0.7)	16.0(0.8)	17.5(0.8)	17.6(1.3)	15.5(1.7)	−0.021	0.09	
Chronic disease (−) (n = 689)	21.9(0.7)	20.2(0.6)	19.4(0.7)	20.7(0.7)	22.0(1.2)	19.7(1.1)	−0.011	0.32	0.35
Chronic disease (+) (n = 586)	21.8(1.0)	21.3(0.9)	21.0(0.8)	19.8(0.7)	20.1(1.0)	18.0(0.9)	−0.033	<0.01	
*p,p′*-DDE									
Age<60 (n = 819)	20.5(0.6)	20.9(0.5)	21.3(0.6)	19.5(0.7)	18.9(1.2)	20.6(1.4)	−0.010	0.37	0.06
Age≥60 (n = 456)	21.7(1.6)	21.4(1.5)	23.0(1.1)	20.2(0.7)	20.2(0.9)	18.1(0.8)	−0.042	<0.01	
White race (n = 675)	24.7(0.8)	24.4(0.8)	25.5(0.7)	23.4(0.6)	23.5(0.9)	21.8(0.8)	−0.018	0.15	0.38
Other races (n = 600)	15.4(0.7)	17.4(0.8)	18.4(0.8)	16.8(0.8)	17.1(1.3)	16.2(1.7)	0.001	0.95	
Chronic disease (−) (n = 689)	20.7(0.6)	20.9(0.6)	21.6(0.7)	19.9(0.8)	18.7(1.2)	20.1(1.2)	−0.011	0.34	0.75
Chronic disease (+) (n = 586)	20.1(1.0)	21.5(1.0)	22.2(0.8)	19.7(0.7)	20.4(0.9)	18.2(0.9)	−0.023	0.07	
β-hexachlorocyclohexane									
Age<60 (n = 819)	20.9(0.6)	20.5(0.5)	20.8(0.6)	19.5(0.8)	21.6(1.5)	19.7(1.2)	−0.009	0.39	<0.01
Age≥60 (n = 456)	23.7(2.0)	19.7(2.0)	22.1(1.1)	21.1(0.7)	20.5(0.9)	17.5(0.8)	−0.055	<0.01	
White race (n = 675)	24.9(0.8)	24.8(0.7)	24.6(0.7)	24.0(0.7)	24.2(1.0)	21.1(0.8)	−0.021	0.12	0.56
Other races (n = 600)	17.4(0.9)	16.5(0.9)	18.2(0.8)	17.1(0.9)	17.5(1.3)	15.0(1.4)	−0.021	0.15	
Chronic disease (−) (n = 689)	20.7(0.6)	20.7(0.6)	21.0(0.7)	20.3(0.8)	22.5(1.7)	18.8(1.1)	−0.009	0.47	0.13
Chronic disease (+) (n = 586)	22.5(1.3)	19.8(1.1)	21.5(0.8)	20.5(0.7)	20.2(0.9)	17.8(0.9)	−0.036	0.01	

*Adjusted for gender, age, race, BMI, smoking status, outdoor activity, and vitamin D supplement.

**Figure 1:**
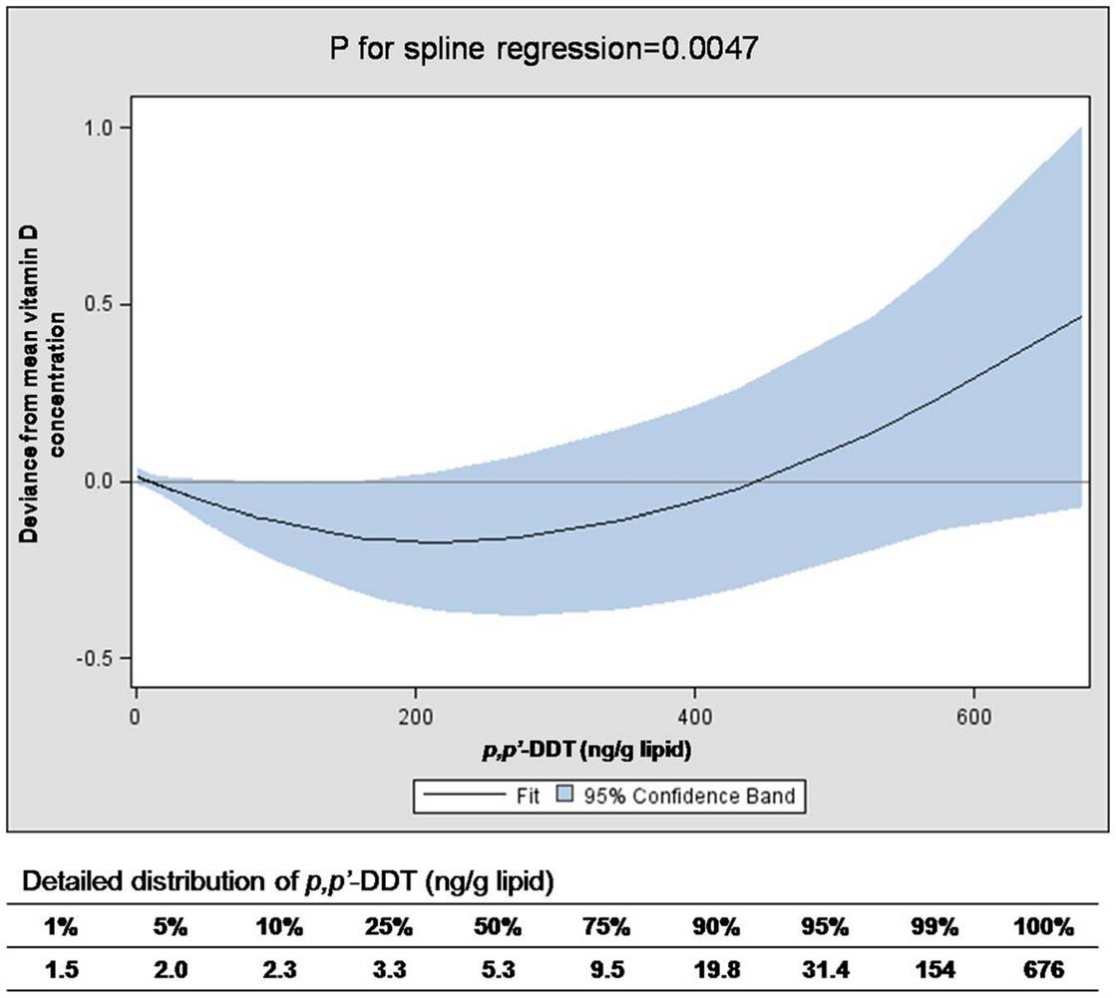
Spline regression of serum concentration of *p,p′*-DDT on serum 25-hydroxyvitamin D (25(OH)D) levels (ng/ml). The curve was adjusted for gender, age, race, BMI, smoking status, outdoor activity, and vitamin D supplement. The distribution of *p,p′*-DDT among study subjects was added below the X-axis. The increasing trend of vitamin D was mainly driven by only a very small number of subjects with very high *p,p′*-DDT.

When we fitted non-linear relationships, the U-shaped pattern was observed with p,p′-DDT (Figure 1). Even though the association between p,p′-DDT and 25-(OH)D appeared to be linear when we used the traditional quintile approach, the linear inverse association persisted only until about 200 ng/g lipid of p,p′-DDT (the 99^th^ percentile was 154 ng/g, as indicated on Figure 1). For higher values, 25-(OH)D appeared to increase, making the U-shaped association.

## Discussion

In this cross-sectional study, we observed significant inverse associations between serum concentrations of several OC pesticides, p,p′-DDT, p,p′-DDE, and β-hexachlorocyclohexane, and serum 25-(OH)D levels among the U.S. general population. To our best knowledge, this is the first study that linked OC pesticides and serum 25-(OH)D levels in human.

In fact, both POPs like OC pesticides and vitamin D deficiency have been associated with a variety of chronic diseases in general populations, but these two important factors have been separately studied by researchers in different fields. As field and animal studies observed that the exposure to POPs can disturb the metabolism of vitamin D [7], [8], [9], we hypothesized that a similar pattern existed in human.

In fact, there were strong positive correlations among serum concentrations of p,p′-DDT, p,p′-DDE, and β-hexachlorocyclohexane (p,p′-DDT vs p,p′-DDE = +0.72; p,p′-DDT vs. β-hexachlorocyclohexane = +0.56; p,p′-DDE vs. β-hexachlorocyclohexane = +0.66). Compared with other OC pesticides evaluated in this study, serum concentrations of these three OC pesticides were more strongly correlated with each other in this population. Therefore, even if there is a causal relationship between vitamin D concentration and one or more POPs, it is difficult to know if all 3 of these OC pesticides are really involved in the disturbance of metabolism of vitamin D based on the epidemiological findings.

Both p,p′-DDT and p,p-DDE are closely related because p,p′-DDT breaks down to p,p′-DDE, an extremely stable compound in the environment, that resists further environmental breakdown or metabolism by organisms [16]. Therefore, even though DDT was banned several decades ago in most developed countries, nearly all U.S. residents have substantial amounts of p,p′-DDE in their bodies. This persisting exposure is owing to the extreme stability of p,p′-DDE and continuous exposure to p,p′-DDE through food consumption, because our current food chain on earth is seriously contaminated with POPs [17]. In this study, stronger and more consistent patterns were observed with p,p-DDT in subgroup analyses as well as analyses based on all subjects than p,p′-DDE. Importantly, DDT is still used for malaria control in Central America, South America, Mexico, Africa and some developing countries in Asia [18]. It may explain why non-white race had a higher concentration of DDT compared with white race.

β-hexachlorocyclohexane is an isomer of technical hexachlorocyclohexane and byproduct of the production of the insecticide Lindane [19]. Technical hexachlorocyclohexane and Lindane were among the most extensively used OC pesticides produced mainly after the Second World War until the 1990s [20]. They have not been produced in the U.S. since 1976, but imported Lindane is available as insecticide or prescription medicine to treat scabies and head lice in humans [19]. Even though India is the only country known where Lindane is currently produced, some developing countries had produced Lindane until very recently [21].

In fact, it is well-known that chronic exposure to DDT causes thin avian egg shells which is one factor in the decrease in population of certain avian species [22]. Thinning egg shells is closely related to calcium metabolism which is largely regulated by two steroids, estradiol and vitamin D [22]. Although it has long been suspected that DDT disturbs the metabolism of vitamin D, early experimental studies in 1960s or 70s failed to show that DDT disturb vitamin D metabolism [23], [24], [25].

However, this issue needs to be reevaluated with low doses of DDT similar to environmental exposure because the doses of DDT used in early experimental studies were very high. If DDT disturbs hormone metabolism, including vitamin D, this disturbance would likely happen due to endocrine disrupting properties rather than toxicity of DDT. In the case of endocrine disruptors, dose-response curves are often nonmonotonic and low dose levels are more relevant to biological effects than high dose levels [26]. In our previous epidemiological studies on POPs in relation to various chronic diseases, we have observed apparent low dose effects of POPs. POPs could be involved in the pathogenesis of these diseases through endocrine disrupting properties of POPs [11], [13].

The results based on traditional linear modeling and category approaches showed a decrease of vitamin D mainly among subjects with the upper 10% of p,p-DDT with the significant p value for linear trend, However, when we fitted the dataset using a General Additive Model, we observed non-linearity of p,p′-DDT, making the U-shaped association. The decrease of vitamin D was mainly observed up until the subjects had p,p′-DDT of 200 ng/g lipid, above which vitamin D showed an increasing trend with the increase of p,p′-DDT. As p,p′-DDT had a very skewed distribution, the interquartile range of p,p′-DDT was only 3.3∼9.5 ng/g lipid, but about 1% of subjects had p,p′-DDT levels ≥154 ng/g and the highest value was 676 ng/g lipid. Therefore, the increasing trend of vitamin D was mainly driven by only a very small number of subjects with very high p,p′-DDT in this sample who had largely background exposure to the pesticide. Similar to our previous findings on POPs [11], [13], this kind of dose-response relation may be explained by endocrine disrupting properties of p,p′-DDT. However, the possibility of increasing trend of vitamin D among persons with high p,p′-DDT needs to be confirmed in further studies with sufficient sample sizes.

There are several limitations. First of all, we cannot rule out reverse causality as this study is cross-sectional. However, we are not aware of mechanisms whereby higher vitamin D level would help to clear serum concentrations of OC pesticides. Also, field findings on POPs and vitamin D levels suggest the possibility that OC pesticides influence vitamin D metabolism. Second, serum concentrations of 25(OH)D are affected by sunlight exposure, and vary by season, time of outdoor activity, and residence latitude. Serum concentrations of 25(OH)D are highest in the summer and the fall and are lowest in the spring. This seasonal variation limits the usefulness of single measurements, and requires an average concentration throughout the year or at least a seasonal adjustment. Unfortunately, we could not make seasonal adjustments or adjust for residence latitude. However, a couple of studies on variation of OC pesticides by region in the U.S. reported the order of concentrations of p,p′-DDE by West>South>Midwest>Northeast, reflecting the history of the geographic manufacture and use of these chemicals [27], [28]. Therefore, the failure of adjusting for living area may lead to underestimation of the association between OC pesticide and vitamin D levels as subjects with high exposure to sunshine are likely to have high exposure to OC pesticides. Finally, we could not exclude the possibility that other POPs not measured in this study could play a role in the pathogenesis of type 2 diabetes, because serum concentrations of POPs are highly correlated in the general population.

If the associations observed here were causal, as evidenced by confirmation in prospective and experimental studies, avoiding exposure to OC pesticides and any efforts to decrease body burden of OC pesticides may be helpful to increase vitamin D levels and decrease vitamin D deficiency-related diseases. There may be other chemicals which also disturb metabolism of vitamin D. Therefore, there should be more human and experimental studies on what kinds of chemicals can cause vitamin D deficiency and what mechanisms would be involved.
